# Childhood atopic dermatitis: current developments, treatment approaches, and future expectations

**DOI:** 10.3906/sag-1810-105

**Published:** 2019-08-08

**Authors:** Pınar GÜR ÇETİNKAYA, Ümit MURAT ŞAHİNER

**Affiliations:** 1 Division of Pediatric Allergy and Asthma Unit, Department of Pediatrics, Faculty of Medicine, Hacettepe University, Ankara Turkey

**Keywords:** Atopic dermatitis, childhood, pathogenesis, treatment

## Abstract

Atopic dermatitis (AD) is the most common chronic inflammatory skin disorder of childhood. Underlying factors that contribute to AD are impaired epithelial barrier, alterations in the lipid composition of the skin, immunological imbalance including increased Th2/Th1 ratio, proinflammatory cytokines, decreased T regulatory cells, genetic mutations, and epigenetic alterations. Atopic dermatitis is a multifactorial disease with a particularly complicated pathophysiology. Discoveries to date may be considered the tip of the iceberg, and the increasing number of studies in this field indicate that there are many points to be elucidated in AD pathophysiology. In this review, we aimed to illustrate the current understanding of the underlying pathogenic mechanisms in AD, to evaluate available treatment options with a focus on recently discovered therapeutic agents, and to determine the personal, familial, and economic burdens of the disease, which are frequently neglected issues in AD. Currently available therapies only provide transient solutions and cannot fully cure the disease. However, advances in the understanding of the pathogenic mechanisms of the disease have led to the production of new treatment options, while ongoing drug trials also have had promising results.

## 1. Introduction

Atopic dermatitis (AD), also known as atopic eczema, is the most common chronic inflammatory skin disorder of childhood and is characterized by pruritus, dryness of skin, and scratching [1]. The disease presents with eczematous, itchy lesions that show distinct distribution in different pediatric age groups, and episodes of clinical exacerbation called flares or flare-ups [1]. AD affects 5%–20% of children [1], and the prevalence differs among geographical regions [2]. The disease usually occurs in early childhood; about 85% of cases are observed during the first 5 years of life [3], and the disease alleviates substantially by the age of 7 [4]. In a small percentage of patients, the disease may begin in adulthood [5]. In addition, AD may be the first manifestation of “atopic march”, which is characterized by development of asthma and allergic rhinitis at a later age [6]. 

AD is a multifactorial heterogeneous disorder that results from the interaction of genetic and epigenetic factors, environmental agents, immunological defects, and epithelial barrier dysfunction [7]. As understanding underlying disease mechanisms is critical for the development of effective treatment strategies, studies focusing on the pathogenesis of AD have increased in recent years [8]. This review focuses on the pathogenesis of the disease and recently discovered novel therapies, in addition to classical treatment methods. 

## 2. Epidemiology

Although AD lesions were well described in antique texts [9], the disease was first described and named by Wise and Sulzberger in 1933 [10]. There are many studies on the frequency and pathogenesis of this common disease; however, the International Study of Asthma and Allergies in Childhood (ISAAC) can be considered the most comprehensive and largest study on AD. According to ISAAC, prevalence of AD varies considerably in different regions [11], and the frequency of childhood AD was reported to range from 0.2% to 24.6% worldwide [1]. Phase 3 of the ISAAC study [12] showed that AD occurs at a higher, relatively stable ratio in developed countries and urban areas, whereas the prevalence is steadily rising in developing countries. Likewise, Civelek et al. reported that the frequency of AD is lower in developing rather than developed Mediterranean countries. In the same study, risk factors and progression of AD also showed differences in developing Mediterranean countries [13]. Compared to ISAAC phase 1, the ISAAC phase 3 study showed stable or decreased AD frequency in developed countries [2], while low-income countries were surprisingly shown to have an increase in frequency. This increase has been attributed to changes in lifestyle [14], as the short time period between phases 1 and 3 would have been insufficient for the emergence of substantial genetic changes [15]. Therefore, it is believed that a modern lifestyle may have caused changes in daily practices, eating habits, and the perception of hygiene among people living in developing countries. The use of household cleaning products containing strong chemicals and increased frequency of soap, shampoo, and shower gel usage are among the factors believed to have contributed to the increased prevalence of AD in these countries [2]. 

## 3. Diagnosis and scoring systems for clinical assessment and severity

AD is diagnosed by clinical examination mainly based on morphological features and distribution of lesions; however, no laboratory or pathological findings specific to the disease have been discovered to date. Therefore, strict application of standard diagnosis criteria is important and even necessary to prevent misdiagnoses of atopic dermatitis in cases of other kinds of dermatitis. Until 1960, there were no criteria for the diagnosis of AD. In 1961 Georg Rajka established the first criteria, followed by Jon Hanifin, who published 13 features for the diagnosis of AD in 1977 [16]. After these initial efforts, Hanifin and Rajka proposed modified diagnostic criteria for AD in 1980 [17]. This modified set of criteria has been shown to have high sensitivity, up to 93% to 96% according to several studies [18,19], and this set of criteria has been used as a basis for later versions of AD diagnostic criteria [20]. According to the original Hanifin and Rajka criteria, a patient was diagnosed with AD when at least 3 of 4 major and at least 3 of 23 minor features were met [16]. However, the minor criteria were not practical to use in clinical practice [21]. The Hanifin and Rajka criteria were used by Kang and Tian (1987, China), Schultz Larsen (1992, Sweden), Japanese Dermatological Association (JDA) (in 1994), Danish Allergy Research Center (DARC) (in 2005), Korean Dermatological Association (KDA) (in 2006), and American Academia of Dermatology in 2003 [22]. Unfortunately, the Hanifin and Rajka criteria were deemed unsatisfactory for epidemiological surveys [20]. Therefore, the United Kingdom Working Party diagnostic criteria (UK criteria, in 1994) [23] and the ISAAC criteria (in 1995) [24] were used for epidemiological studies. Although the UK criteria were appropriate for clinical practice, they were not applicable for small children, especially infants. The ISAAC criteria have been utilized as the gold standard diagnostic criteria in global surveys for all childhood age groups since the 1990s [20,25]. 

Assessment of disease severity is particularly important in clinical practice when determining appropriate treatment for the patient, identifying the time of treatment cessation, and for epidemiological surveys. For this purpose, a comprehensive scoring system was published in 1993 based on a consensus by the European Task Force Group on Atopic Dermatitis (ETFAD), with participation of over 20 experts [26]. This scoring system was named SCORAD, an acronym for “*SCOR*ing *A*topic *D*ermatitis” [26]. This scoring system comprises both objective (A. Extent: area involved according to the rule of nines; B. Intensity: erythema, edema/papules, scratching, crust formation, lichenification, and dryness) and subjective symptoms (C. Pruritus and loss of sleep) [26], and total score is calculated with the formula A / 5 + 7B / 2 + C [26]. The final score is extremely variable and is highly dependent on the physician’s examination. Consequently, an objective SCORAD which omits the subjective C criteria was put forth in 1997 [27]. Other than these scoring systems, a simplified score named Three-Item Severity (TIS) which evaluates erythema, edema, and excoriation [28], SASSAD (Six Area, Six Sign Atopic Dermatitis) that encompasses 6 signs (erythema, exudation, excoriation, dryness, cracking, and lichenification), each on a scale of 0 (absent), 1 (mild), 2 (moderate), or 3 (severe), at each of 6 sites (arms, hands, legs, feet, head and neck, and trunk) [29]; finally, the EASI (Eczema Area and Severity Index) has been developed [30]. Nevertheless, TIS is less sensitive than SCORAD [31]; SASSAD does not have the parameters of pruritus and loss of sleep and has disadvantages in calculation of an objective score; EASI scoring is a time-consuming and rather complex system [32]. Nowadays, objective SCORAD has been widely used and is considered the most accurate scoring system for AD [32]. 

AD may be confused with many other skin diseases, and differential diagnosis should be made with these disorders. Several diseases, including diseases causing immunodeficiency, may present with eczematous skin rash and could mimic AD. Seborrheic dermatitis, especially in infancy, dermatitis herpetiformis, irritant contact dermatitis, nummular dermatitis, and psoriasis are common inflammatory skin disorders that are often mistaken for AD [33,34]. Other skin conditions which may be ascribed to AD include allergic contact dermatitis, scabies, molluscum contagiosum, tinea corporis and capitis, mycosis fungoides, Langerhans cell histiocytosis, and pityriasis lichenoides chronica [35]. Additionally, a long list of immunodeficiency disorders, such as hyperimmunoglobulin E syndrome, Wiskott–Aldrich syndrome, Netherton syndrome, immunodysregulation polyendocrinopathy enteropathy X-linked (IPEX), severe combined immunodeficiency (SCID), and Omenn syndrome may be diagnosed in children who present with symptoms suggestive of AD [33,36]. 

## 4. Novelties in pathogenesis of atopic dermatitis

**Figure 1:**
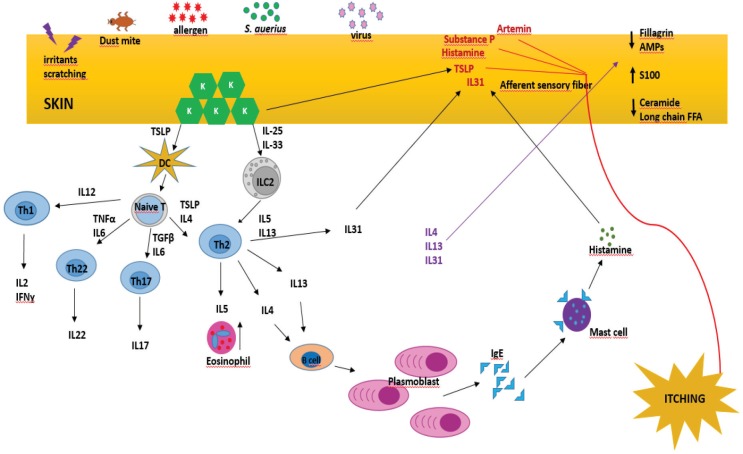
Overview of the pathogenesis of atopic dermatitis [254].

AD is a multifactorial and heterogeneous skin disorder resulting from genetic predisposition, environmental factors, skin barrier defects, and immunological abnormalities [8] (Figure 1). Elucidating the underlying pathogenesis and understanding the mechanism that causes itching are essential for determining the most effective treatment.

### 4.1. Epithelial barrier dysfunction

There are many molecules in the structure of the epidermis that are critical in preventing water loss and providing protection against environmental factors [37]. Mutations in the genes encoding for proteins responsible for normal barrier function are among the main reasons for AD development [37]. Defined mutations are located in filaggrin (FLG), desmoglein-1 (DSG1), corneodesmosin (CDSN), serine protease inhibitor Kazal-type 5 (SPINK5), Matt [38], and lymphoepithelial Kazal-type–related inhibitor (LEKTI) [37]. The SPINK5 gene encodes LEKTI which is involved in the pathogenesis of AD-like disorders, such as Netherton syndrome, a syndrome characterized by severe dermatitis, allergic diseases, and high serum IgE levels [39]. Skin barrier integrity is maintained by fillagrin via keratinocyte formation. Degraded end products of fillagrin maintain water balance, low acid pH, and barrier function of the skin [40]. Decreased or defective fillagrin molecules lead to high pH and enhance the function of serine protease kallikrein (KLK) [41]. KLK binds to its receptor and thus induces the production of thymic stromal lymphopoietin (TSLP), which promotes inflammation [42]. Inflammation causes further degradation of fillagrin end products and decreases fillagrin production [43]. As a result of decreased fillagrin and its end products, allergen penetration is enhanced [44], as well as bacterial colonization [45]. Changes in the lipid composition of the stratum corneum [46] and looseness of tight junctions are other factors that substantially contribute to AD pathology [47]. Tight junction defects and the claudin-1, claudin-23, and ZO-1 molecules have been associated with skin problems in AD [47,48]. Additionally, polymorphisms in the CLDN-1 gene which encodes claudin-1 have also been linked to AD pathology [47].

Sensitization with food allergens can occur not only via the gastrointestinal tract but also through defective and inflamed skin even before the ingestion of the allergen foods [49]. By this route, 35% of the children with AD sensitize to foods by the age of 2 years [50]. The association between inhalant allergens and AD has been demonstrated in about 65% of patients at the age of 6 years old [51].

### 4.2. Skin microbiota

Many bacteria and fungi colonize on the skin; however, in AD, the bacterial composition of skin completely changes due to an increased adhesion of various bacteria to the skin of those with AD [52]. *Staphylococcus aureus* occurs at up to 90% of lesion sites [53], while it is found at lower percentages in healthy skin. IL-4, an important cytokine of the Th2 pathway, is crucial for adhesion of *Staphylococcus aureus* onto skin [54]. Staphylococcal α-toxin induced keratinocyte death was shown to be increased by IL-4 and IL-13 through activation of signaling transducer and activator of transcription 6 (STAT6) [55]. It has also been shown that toxins of the bacteria increase inflammation in the skin, thereby exacerbating symptoms [56]. Fillagrin has a neutralizing effect on *Staphylococcus aureus* α-toxin in healthy skin [57], and fragmented filaggrin products inhibit the growth of *S. aureus* [58]. Additionally, enterotoxin B of the bacteria induces the production of IL-31, one of the major cytokines responsible in pruritus, amplifying the severity of symptoms [59].

Lastly, the increase in fibronectin deposition in the stratum corneum of patients with AD serves as another mechanism that enables adhesion of *Staphylococcus aureus *in the epidermal layer [60]. Although sphingosine prevents the colonization of the bacteria in normal healthy skin, its levels are diminished in lesion sites [61]. Furthermore, the high pH, water loss, and dryness that are characteristic of the disease are known to ease the adhesion of *Staphylococcus aureus *to skin [61]. 

### 4.3. Immunological mechanisms underlying AD

Immunological background is rather complicated in AD; however, cytokine profile has been shown to have an important effect on the life cycle of keratinocytes [62]. Keratinocytes express pattern recognition receptors including Toll-like receptors (TLRs), NOD-like receptors (NLRs), C-type lectin receptors (CTLRs), and RIG-I-like receptors (RLRs) [63]. These receptors recognize pathogenic antigens, subsequently causing an increase of proinflammatory cytokines [63]. Cells receive signals from their environment and transduce these inputs through intracellular signal molecules. When a relevant signal arrives to the cell surface, the cell responds by producing intracellular cyclic adenosine monophosphate (cAMP) and cyclic guanosine monophosphate (cGMP), which increase the phosphorylation of protein kinase A (PKA). This pathway leads to a decline in the levels of proinflammatory molecules such as IL-2, IL-4, IL-6, IL-10, and TNF-α, thereby suppressing innate immunity [64]. Phosphodiesterase type 4 (PDE4) inhibitors prevent cAMP degradation [65] and can therefore be used to reduce the levels of inflammatory cytokines [66]. On the other hand, keratinocytes contribute to innate immunity by producing antimicrobial peptides (AMPs) (defensin, dermicidin, cathelicidin, calprotectin) which damage microbial cellular membranes [67]. Abnormalities in the production of these molecules may contribute to worsening of AD.

In patients with AD, cytokine profile shifts from T helper 1 (Th1) to T helper 2 cells (Th2). In the acute phase of AD, there is an excess of Th2 cytokines including IL-4, IL-5, IL-9, IL-13, IL-31 [68], as well as IL-22, which is also produced by Th22 lymphocytes [69], a type of lymphocyte which is crucial in epidermal immunity. These cytokines upregulate inflammatory status and cause overproduction of immunoglobulin E; IL-22 is known to inhibit keratinocyte differentiation [70]. IL-22 is also responsible for skin barrier integrity, and its level has been shown to be correlated with AD severity [71]. TSLP, an IL-7–like cytokine, is expressed by epithelial cells and binds to antigen-presenting cells (APCs) including dendritic cells, macrophages, monocytes, and T and B lymphocytes in the epidermal layer of the skin [72]. Moreover, TSLP increases IL-4 levels, which then further increases TSLP production [73]. Keratinocytes in lesion sites express TSLP, IL-25, and IL-33, which increase Th2 cytokines by activating dendritic cells (DCs) in the skin [74]. These stimulated DCs then migrate to regional lymph nodes and are instrumental in the conversion of naïve T cells to Th2 lymphocytes [75]. Furthermore, TSLP, IL-25, and IL-33 promote the production of innate lymphocyte group 2 cells (ILC2), increase their response, and induce the production of cytokines, including IL-5 and IL-13 [74]. On the other hand, ILC2s elicit IgE isotype class switching in cells [76]. IL-4 and IL-13 are also responsible in the chronic phase through their contribution to tissue remodeling [77]. IL-5 is primarily produced by Th2 and mast cells and participates in eosinophil growth, chemotaxis, and survival [78]. Recent studies have shown that IL-31 has a substantial effect on the development of pruritus [79]. IL-31 is mainly produced by Th2 lymphocytes and to a lesser extent by dendritic cells upon activation [80]. Intriguingly, eosinophils in patients with AD express IL-31 more intensely compared to healthy individuals [81]. IL-31 binds to its receptor IL-31 receptor RA (IL31RA) on sensory nerves and induces an itching sensation, as well as facilitating the elongation and branching of sensory nerves [79]. 

Th17 lymphocyte is another cell type that contributes to the acute phase [62]. This cell produces IL-17 and IL-22, which increase proinflammatory molecules, S100 proteins, and other types of AMPs in keratinocytes [82]. S100 proteins act as AMP and damage-associated molecular pattern molecules, which initiate the inflammatory cascade [83]. The level of S100 protein increases in acute and chronic AD; thus, it is thought to be an important parameter in preserving enhanced inflammatory status [83]. Furthermore, increases in IL-17 lead to eosinophil- and neutrophil-mediated inflammation [84], while reduced IL-17 levels are correlated with reduced AMP, which has been reported to be associated with increased susceptibility to skin infections [85]. In the chronic phase, interferon gamma (IFNɣ), a Th1 cytokine, is believed to be more prevalent, along with Th2 and Th22 cell responses [86].

## 5. New insights in the mechanism of pruritus

Cytokines and other chemical molecules that cause itching (pruritogens) are released from eczematous skin areas where they activate the relevant sensory nerves and cause pruritus; thus, the patient feels the need to scratch these areas [87]. Hyperesthesia in AD is due to aberrantly elongated sensory nerves in the upper layer of the skin which are increasingly exposed to environmental factors such as dryness, irritation, and chemicals [88]. Histamine, substance P, TSLP, and IL-31 act on these elongated nerve fibers, contributing to the itching sensation [89,90]. Heat is an important aggravating factor for itching in AD [91]. Artemin (enovin, neublastin) is a substance which has been shown to increase in AD lesions and is reported to cause itching, especially in warm temperatures [92].

Pruritus has detrimental effects on sleep and may reduce the length and quality of sleep, which leads to a decrease in melatonin levels. Decreased melatonin levels have been suggested to promote further itching [93]. Additionally, decreased cortisol level and increased inflammatory status due to elevated IL-2 levels are other factors that contribute to itching at night [93]. Sweating is another factor that exacerbates itching in AD, due to the normal effects of excess sweat on the skin and also the abnormal composition of sweat in patients with AD [94]. Conversely, keratinocyte debris may obstruct sweat glands and prevent normal sweating; this also leads to raised body temperature and could increase pruritus [95].

## 6. Genetic and epigenetic factors

Fillagrin mutation is the most important predisposing genetic change in AD. However, it is found in only 10%–50% in patients with AD, and in 9% of the healthy population [69]. Therefore, AD cannot be explained only by Fillagrin mutation. Genome‑wide association studies (GWAS) have been initiated to elucidate the pathogenesis of AD. Many novel genes have been identified in patients with AD, especially after the first GWAS published by Esparza-Gordillo et al. in 2009 [96]. Genes responsible for the pathogenesis of AD are given in Table 1. TRAF6, RAG1, RAG2, SOCS1, NGFR, 4q27 IL2/IL21, 11p13 PRR5L, 16p13.13 CLEC16A/DEXI, and 17q21.32 ZNF652 are the novel genes that have been reported in GWAS studies on AD [97]. Additionally, several genetic polymorphisms have been found to be related to AD [98]. These include polymorphism of vitamin D pathway molecules [99] and polymorphisms of cytokines IL-4, IL-5, IL-9, IL-13, and IL-31, as well as their receptors [100]. Aside from genetic mutations, epigenetic mechanisms have been implicated in AD pathogenesis [101]. Epigenetic mechanisms regulate expression of genes without altering the DNA sequence [102]. These mechanisms are mainly DNA methylation, microRNA (miRNA), and histone tail modification [102]. It has been shown that epigenetics affect gene expression through environmental factors such as pollutants, tobacco smoke, aging, and diet [103]. DNA methylation differences between normal epidermis and AD epidermis have been shown in a study focusing on epigenetic factors [104,105]. miRNAs have fundamental roles in cell proliferation, differentiation, apoptosis, signal transduction, and organ development [106]. miRNAs are upregulated in the skin of patients with AD [107]. Children who were exposed to smoking during pregnancy have high miRNA-223 and low T regulatory (Treg) lymphocytes [108]. These children were found to have a higher tendency to develop AD during the first 3 years compared to children without exposure to smoking during pregnancy [108].

**Table 1:** Genetic mutations underlying pathogenesis of atopic dermatitis.

Gene symbol	Gene name	Chromosomal location	Genetic variation	Disease severity	References
Epidermal differentiation					
FLG	Fillagrin	1q21.3	R501X, 2282del4	correlated with severity	[69]
FLG2	Fillagrin 2	1q21.3	rs12568784, Q2053del224 rs16833974	persistent disease	[222]
SPINK5/LEKTI	Serine protease inhibitor Kazal type 5/Lymphoepithelial Kazal type related inhibitor	5q31	rs2303070 T, E420K	increased disease risk	[223,224]
TMEM79	Transmembrane protein 79	1q22	rs6684514	increased risk of AD	[38]
claudin-1		3q28	rs893051, rs9290929	Mold infection	[225]
MHC (HLA)	Major histocompatibility complex (Human leucocyte antigen)	6p21	TAP1 (Val333IIe, Gly637Asp) TAP2 (IIe379Val, Thr565Ala, Ala665Thr, Gln687Stop)		[226,227]
Innate immunity					
TLR2		4q32	rs4696480, rs3804099, rs3804100, rs5713708	severe disease	[228–230]
TLR4		9q32/33	rs4986790, rs4986791, rs2770150	increased risk of AD	[228,230,231]
TLR6		4p14	rs5743810		[148]
TLR9		3p21.3	C-1237T		[130]
NOD1(CARD4)	Caspase recruitment domain-containing protein 4	7p14/15	rs2907748, rs2907749, rs2736726, rs3030207, rs2075818	allergen sensitization	[232,233]
CARD 11	Caspase recruitment domain-containing protein 11	7p22.2	p.Glu57Asp (E57D) p.Leu194Pro (L194) p.Arg975Trp (R975W) p.Met183_Lys196	severe disease	[234]
CARD 14	Caspase recruitment domain-containing protein 14	17q25.3	rs11652075		[235]
NOD2 (CARD15)	Caspase recruitment domain-containing protein 15	16q21	rs1077861		[232,236]
TSLP	Thymic stromal lymphopoetin	5q22	rs1898671	eczema herpeticum	[237]
DEFB1	Human β-defensin 1	8p23	rs5743399, rs5743409		[238]
Adaptive immunity					
IL-4	Interleukin 4	5q31-33	590C/T, 1098G/T, 589C>T	increased risk of AD	[100,239–241]
IL-4Ra	Interleukin 4 receptor alpha	16	I50V, Q576R	increased risk of disease	[100]
IL13	Interleukin 13	5q31.1	rs12188917	association with asthma	[98]
IL-31	Interleukin 31	12	1066, _2057, and ivs2 +12	high intensity of pruritus	[242]
IL-17A	Interleukin 17		152 G/A	association with asthma and severe disease	[243]
IL9	Interleukin 9	5q31/35	4091G>A		[244]
IL9R	Interleukin 9 receptor	Xq/Yq	1737C>T		[244]
IL18	Interleukin 18	11q22.2/3	132A>G, 133C>G, 137G>C, 113T>G, 127 C>T		[245]
GM-CSF	Granulocyte macrophage colony stimulating factor	5q31.1	677A>T, 1916T>C		[246]
Chemokines and related genes					
CCL5 (RANTES)	Chemokine (C-Cmotif) ligand 5 (Regulated upon activation, normally T cell expressed + secreted)	7q11.2/q12	28C>G, 403G>A, 2518 A>G	allergen sensitization	[247,248]
CCL11 (eotaxin 1)	Chemokine (C-Cmotif) ligand 11	17q21.1/q21.2	426C>T, 384A>G		[249]
CCL17 (TARC)	Chemokine (C-Cmotif) ligand 17 (thymus and activation-regulated chemokine)	16q.13	431C>T		[250]
Vitamin D pathway					
Cyp24a1		20q54	rs2248359	severe disease	[251]
VDR		20q13	rs7975232	severe disease with high eosinophil and IgE levels	[99]
Other genes					
CTLA4	Cytotoxic T lymphocyte associated antigen-4	2q33	49A>G		[252]
IRF2	Interferon regulatory factor 2	4q35	829C>T, 830C>T, 684C>T		[253]

## 7. Current treatment of atopic dermatitis

### 7.1. Moisturizers and bathing

**Figure 2:**
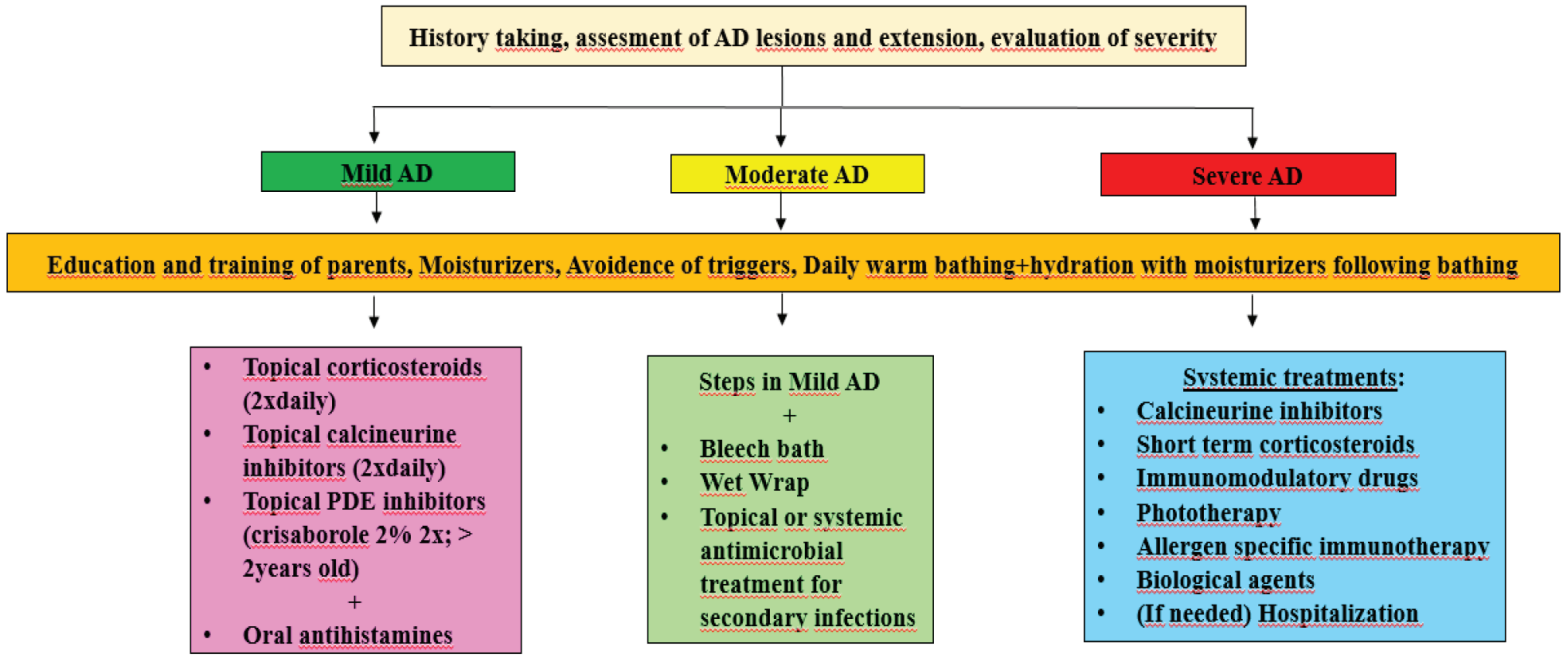
Treatment algorithm of atopic dermatitis in children [113,255,256].

Treatment of AD is aimed at suppressing inflammation, eliminating identified triggers, reducing pruritus, and combating the development of xerosis, a major feature of AD [109,110] (Figure 2). For this purpose, conventional therapy is the first-line treatment approach. However, new therapeutic strategies and agents are continuously being developed with the help of advances in the understanding of AD pathophysiology. Basically, topical moisturizers are the mainstay of AD treatment, as they prevent dehydration, soften the skin, and increase water retention. Above all, they serve to minimize the need for pharmacological drugs for AD [110,111]. Moisturizers are the first line of therapy in mild AD and constitute an important and primary step in moderate and severe forms of AD [112]. Moisturizing the skin with different types of emollients, occlusive agents, and humectants decrease inflammation, pruritus, and xerosis, and also reduce the requirement for topical steroids and antibiotics [110]. There are many types of products in the form of ointments, lotions, creams, emollients, oils, and gels. Even though there are some differences with regards to their contents, the most important aspect of these moisturizers are to ensure that they are perfume- and fragrance-free and do not contain harsh chemicals [110]. In addition to moisturizers, daily bathing with warm water is an extremely important step for hydration of skin, as well as removing allergens, irritants, crusts, and bacteria [113]. Applying moisturizing agents immediately after bathing has been a fundamental approach for treatment in patients with AD [113]. Otherwise, bathing without moisturizing has a negative impact on AD. 

### 7.2. Wet-wrap and bleach therapy

Wet-wrap therapy in combination with topical agents is an effective method used in the treatment of AD flare-ups [114]. Generally, topical agents are applied on the skin followed by a layer of tubular wet bandage and an outer layer of dry bandage [110]. This technique intensifies the effect of moisturizers by providing a smooth skin texture and preventing water loss [110]. A bleach bath was thought to be effective in the treatment of AD, because some physicians reported that eczematous lesions benefited from the chlorinated water of swimming pools [110]. Additionally, Huang et al. showed the efficacy of bleach bath combined with intranasal mupirocin treatment twice a week compared to placebo [115]. Bleach baths have been considered to reduce the colonization of *Staphylococcus aureus* on the skin, preventing secondary skin infections [116]. In this therapy, the child is soaked in water containing a certain amount of bleach (1/4 cup of bleach into 35–40 L of water) for 5 to 10 min [117,118]. However, bleach baths were shown to be ineffective in the treatment of AD in some studies [119]. Other than bleach or sodium hypochlorite, there are antiseptic agents including triclosan, potassium permanganate, and chlorhexidine gluconate used for both the management of infected skin and the prophylactic treatment of AD. Bathing with antiseptic agents has been shown to be useful in diminishing the bacterial load on the skin of patients with AD. Furthermore, no significant effect on reducing bacterial load on inflamed skin in AD has been identified in a few studies [120]. Therefore, combination therapy of antibiotics along with antiseptic agents has been shown to be particularly effective in the treatment of clinically infected skin in AD [115]. 

### 7.3. Topical antimicrobials

AD exacerbations and acute flares are mostly associated with bacterial and viral infections, including *Staphylococcus aureus*, *Streptococcus pyogenes*, *Herpes simplex*, *Varicella zoster* viruses, papillomavirus, and molluscum contagiosum [121,122]. *S. aureus* mostly colonizes the skin and the nasal mucosa, and is frequently associated with worsening of the eczematous lesions [122]. Mupirocin and fusidic acid are effective antibiotics for the prevention of *S. aureus* colonization when used twice a day for a week [123,124]. Furthermore, application of mupirocin twice a day in both nasal orifices for 5 days in a month for 3 to 18 months has been shown effective in eradicating *S. aureus* [124]. Systemic antibiotics are given in the presence of clear evidence of bacterial infection, not to prevent colonization [125,126]. The first choice of antibiotic treatment is beta-lactam antibiotics for 7–14 days [127].

About 3% of AD cases are prone to severe viral infections, especially *Herpes simplex* and *Varicella zoster* viruses even after immunization [111,122]. The clinical picture caused by *Herpes simplex* virus is eczema herpeticum, which requires administration of systemic acyclovir for treatment [128]. Prompt initiation of acyclovir treatment significantly reduces the mortality of eczema herpeticum [128]. 

## 8. Topical antiinflammatory therapy

Antiinflammatory therapy with topical calcineurin inhibitors (TCI), including tacrolimus and pimecrolimus, as well as topical corticosteroids (TCS), is reported to be the most effective approach in the treatment of acute flares [118]. Excessive inflammatory response in the skin is minimized by antiinflammatory treatment agents during flares [118]. Both TCS and TCI suppress T lymphocytes and inhibit proinflammatory cytokine release from immune cells [110,129]. Therefore, these agents are useful in controlling inflammation, pruritus, and skin eruptions [118]. 

## 9. Topical corticosteroids

TCSs are widely used; they are the first-line treatment for acute flares in AD [110,130]. They are indicated for eczematous lesions unresponsive to daily skin care and proper usage of emollients, creams, and ointments [116]. TCSs exhibit their effect by inhibition of T lymphocytes [110], thus reducing inflammation on the skin; they are also known to abate pruritus. In addition, topical corticosteroids are not only used for acute attacks but also for prevention of relapses [110]. 

TCSs are divided into 7 categories according to their potency [110]. Corticosteroids with higher potency should be applied for acute flares, whereas low potency corticosteroids are highly recommended for chronic long term usage [110,131]. The optimal suggested period of application is twice daily for 2 weeks, and only on affected surfaces of the skin for protection from local adverse effects of corticosteroids [116]. Many local side effects have been reported, including hypertrichosis, striae, telangiectasia, and skin atrophy for long term skin application, as well as glaucoma and cataract after use on the periorbital region [116]. Therefore, TCIs are recommended for thin skin surfaces such as eyelids and periorbital areas [132]. Studies have demonstrated that skin atrophy is extremely rare in children [133]. Systemic adverse effects, including the suppression of the hypothalamic–pituitary–adrenal (HPA) axis, have been reported after use of high potency topical corticosteroids in children [134]. Use of low to moderate potency topical corticosteroids for 3 to 4 weeks has been demonstrated to rarely affect the HPA axis [134]. Additionally, the possibility of growth retardation in children has been researched in many studies; however, results have mostly revealed only insignificant delays in growth [135]. 

## 10. Topical calcineurin inhibitors

Tacrolimus and pimecrolimus were approved for AD treatment in 2000 and 2001, respectively [136]; they are safe for use in adults and children over 2 years old [136]. TCIs are a product of the *Streptomyces* genus that inhibits calcineurin-dependent T-cell activation and proinflammatory cytokines. They exert their effects by inhibiting mast cells, dendritic cells, and T lymphocytes [137]. In studies, TCIs have been shown to be more effective than mild TCSs and as effective as moderate TCSs [111,138,139]. Therefore, TCIs are suggested for severe, TCS-unresponsive AD lesions, and also for sensitive areas such as eyelids and the face, and when concerned about steroid-related adverse effects [132,140]. However, erythema, burning, and itching sensations have been frequently reported with TCIs [141]. Additionally, many carcinogenic effects were reported during trials on animals [136]. Systemic absorption of TCI has been shown in patients with AD through the disrupted skin barrier and other skin areas [136]; therefore, side effects may be seen more frequently in pediatric age groups [136]. Pimecrolimus 1% and tacrolimus 0.03% are approved for children who are between 2 and 15 years old; 0.1% ointment of tacrolimus is not allowed for pediatric patients [110,136]. Both pimecrolimus and tacrolimus are contraindicated in acute viral skin infections, suspicion of skin malignancy, or immunodeficiency [136]. Although the exact relationship between malignancy and calcineurin inhibitors cannot be demonstrated, skin tumors and lymphoma have been reported [142]. Therefore, long-term use of these agents in all age groups should be avoided. In clinical trials, tacrolimus and pimecrolimus have been shown to be effective on moderate to severe and mild to moderate AD, respectively [143]. TCI should be used twice a day over 2 to 3 weeks, then should be reduced to once a day for 2 weeks [144]. At the beginning of the treatment, burning and itching are significant problems for patients and may reduce treatment compliance. Concomitant steroid therapy may overcome this issue [144]. Although safe for use and very effective, there is a risk for rebound after the cessation of therapy with TCIs; in the event of a rebound or flare-up, TCIs may be restarted twice a day again [144]. 

## 11. Systemic treatment

Systemic therapy for AD is considered when the skin lesions do not ameliorate after usage of topical treatments [145]

### 11.1. Systemic corticosteroids (SCS)

SCS therapy has many side effects, and therefore it is applied for only a week, especially in adults [146]. It has little efficacy on improvement of lesions, and its adverse effects usually outweigh its benefits [147]. Cushing syndrome, hyperglycemia, osteoporosis, and peptic ulcer are primary side effects of systemic steroid therapy [148]. Due to these adverse effects, they are not recommended for routine treatment and recommended for use only for a short time until symptoms are alleviated in severe cases [145,149,150]. 

### 11.2. Cyclosporine 

Cyclosporine exhibits its effect by inhibiting calcineurin receptors which induce the proliferation of IL-2 cytokine. This cytokine is vital for T helpers, T regulatory lymphocytes, Natural Killer cells, and monocytes. By blocking the production of IL-2, the activity and proliferation of T lymphocytes are hampered [151]. Patients with severe, topical therapy-resistant lesions have been shown to benefit from oral cyclosporine treatment [152]. Recommended dosage is 2.5 mg/kg/day in 2 divided doses, not exceeding a maximum dose of 5.0 mg/kg/day [153]. Serum levels of cyclosporine should be monitored routinely and managed according to clinical symptoms and serum levels of the drug. If there is clinical advancement, duration of the therapy may be extended to 12 months [151]. In case of the clinical improvement, cyclosporine therapy is reduced and terminated in 2–3 months [151]. Most frequently observed side effects are headache, hypertension, renal dysfunction, hypertrichosis, hepatotoxicity, and gingival hyperplasia in adults [152]. However, these are extremely rare in children [154]. Cyclosporine has been well tolerated by children and is particularly effective in this age group [155]. 

### 11.3. Azathioprine

Azathioprine acts as an immunosuppressant on proliferating cells. It is a purine analogue inhibiting DNA/RNA synthesis and prevents proliferation of both T and B lymphocytes [156]. Azathioprine has been used with substantial benefits for AD treatment in adults and children [156]. Azathioprine is generally used for a short period in patients who are refractory to cyclosporine [155]. Its effect arises 2–4 weeks after initiation of the therapy, and it is thus considered to be a secondary preference for treatment of AD [149]. The most substantial adverse effects of azathioprine are hepatotoxicity, increased risk of malignancy, and myelosuppression [157]. The recommended dose is 2–4 mg/kg/day, and whole blood count should be monitored for cytopenia regularly [158]. 

### 11.4. Methotrexate 

Methotrexate is a folic acid antagonist which inhibits cell division and DNA/RNA synthesis and suppresses the immune system, which are similar to the effects of azathioprine [149]. The efficacy of the drug has been shown in children and it is well tolerated in the pediatric age group [159]. It has been suggested to be effective in improving skin lesions with few side effects and is considered a safe option for children [159]. However, it may cause bone marrow suppression, increase transaminase levels, and result in cytopenia. Furthermore, although extremely rare, pulmonary fibrosis has been shown to be a complication of the drug [149]. Beginning of action may be prolonged up to 3 months, as is sometimes the case with azathioprine [149]. There is little data on the application of methotrexate in children. In reports, a low dose (5–15 mg/week orally) has been demonstrated to be effective in alleviating lesions [159,160]. 

### 11.5. Mycophenolate mofetil (MMF)

MMF is an immunosuppressive drug derived from mycophenolic acid. It regulates purine synthesis and inhibits T and B lymphocytes [161]. MMF was used in adult patients for the first time in 1999 [162], and was applied to children over 2 years old with refractory AD in 2007 [163]. Pediatric results were promising and no significant undesirable effects were reported. The most frequent adverse effects seen in children were nausea, vomiting, and diarrhea [163]. In addition to these side effects, hepatotoxicity, myelosuppression, and susceptibility to infections may be seen during treatment [163]. Daily recommended dose is 600 mg/m2/day in 2 divided doses in children [164]. 

## 12. Other therapies

Extracorporeal photopheresis and intravenous immunoglobulin therapies have been applied to patients with AD [165,166]. There are limited data available about these methods, and results of studies which utilized these methods showed that they had little efficacy on AD. 

### 12.1. Antihistamines

Itching is the most intolerable symptom of AD and it has devastating effects on social life and sleep. First-generation antihistamines may be beneficial due to their sedation properties [167], but second-generation antihistamines have less sedative effect [167]. Therefore, only first-generation drugs may be used for severe cases of pruritus and overcoming sleep disturbance.

### 12.2. Allergen immunotherapy

Atopy is defined in up to 70% in patients with AD and exposure to aeroallergens have been shown to cause acute flares [130,168]. Removal of allergens from patients with AD with underlying etiology of allergy is an important step in treatment, and avoidance of aeroallergens has been suggested in the literature to decrease flare-up frequency and the severity of AD [130]. There are many studies on immunotherapy and AD; however, outcomes of the reports are conflicting, and contrary to studies on allergic rhinitis, efficacy is questionable in AD [169]. Atopic march, a severe problem in children with AD, should be prevented; however, the preventive role of allergen immunotherapy has not been demonstrated yet [170]. Nevertheless, patients with severe AD and accompanying allergic rhinitis and/or asthma may be considered for allergen immunotherapy [171]. 

## 13. Biological agents and new treatment strategies

### 13.1. Interferon gamma (IFNɣ)

IFNɣ inhibits T helper 2 lymphocytes and reduces IgE production [172]. In clinical trials, patients with moderate to severe AD have been shown to benefit from IFNɣ therapy [173,174]. The dose used in the pediatric age group was 50 µg/m2 for 22 months; clinical improvement was observed [173,174]. However, a specific recommended dose for IFNɣ for AD has not been determined and the Food and Drug Administration (FDA) has not approved the use of IFNɣ for AD [172]. 

### 13.2. Omalizumab

Omalizumab is a recombinant humanized mAb (rhmAb) that inhibits binding of free, unbound IgE to the high-affinity IgE receptor (FcεRI) on mast cells and basophils [175]. Its first use for severe asthma was assessed in the Global Initiative for Asthma Guidelines study in 2003 [176]. Furthermore, omalizumab was initially used on children who were between 6 and 12 years old in 2009 for severe asthma [177]. Later, Barrios et al. reported the efficacy of omalizumab in their pediatric group of patients with AD in 2013 [178]. Doses were not standardized and varied from 150 mg/dose to 375 mg/dose every 2 to 4 weeks in various studies [179,180]. The drug was well tolerated in children without any particular side effects, but clinical trials on the use of omalizumab for AD are very few, and they report debatable efficacy [181,182]. 

### 13.3. Rituximab

Rituximab is an anti-CD20 monoclonal antibody. It was shown to be beneficial on severe AD lesions in 2008 [183]. However, that was followed by a report on the clinical experience of 2 patients with severe AD who had only minimal transient clinical benefits from rituximab therapy [184]. There is no standardized recommended dose for the usage in patients with AD and further studies with higher numbers of patients are required to evaluate its efficacy in AD. 

### 13.4. Dupilumab

Dupilumab is a human monoclonal antibody against a subunit of the IL-4/13 receptor [185]. In a 2014 clinical trial, significant improvements in patients were observed at the end of the 3-month treatment period [186]. The FDA approved dupilumab for use in adults with moderate-to-severe AD in March 2017 [172]. Clinical trials on children are ongoing [172]; preliminary results indicate that results with dupilumab could be effective in the treatment of AD. The dose of the drug was 2 mg/kg in patients aged 6–11 years and 4 mg/kg in patients aged 12–17 years, or 200–300 mg at intervals of 2–4 weeks in clinical trials [187]. 

### 13.5. Mepolizumab

IL-5 is critical for eosinophil growth and differentiation [188]. Eosinophils are essential contributors to AD pathophysiology and have been shown to be elevated in the serum of patients with AD [188]. Therefore, it was suggested that mepolizumab, a human IL-5 mAb, may have an important effect on the treatment of AD [172]. However, initial studies in 2005 did not yield positive results and no clinical improvement was seen [172,189]. 

## 14. Biological agents in trials

Biological agents are new therapeutic drugs targeting the molecules responsible in the pathogenesis of disease*. *Omalizumab, rituximab, interferon gamma, and mepolizumab have already been tried, while new agents that function by affecting the various stages of pathogenesis are under ongoing investigation. The primary agents among these new potential treatments are nemolizumab, ustekinumab, tralokinumab, lebrikizumab, antithymic stromal lymphopoietin receptor (TSLPR), and phosphodiesterase (PDE) inhibitors.

### 14.1. Nemolizumab

Nemolimumab (CIM331), a humanized anti-IL-31 receptor A mAb, binds to IL-31 receptor A, therefore inhibiting IL-31 signaling [190]. This promising agent is thought to be especially effective for pruritus, and therefore may alleviate problems such as sleep disturbance and psychological concerns in both patients and parents. In clinical trials, pruritus has been reported to be reduced 2-fold compared to placebo [190,191]. 

### 14.2. Ustekinumab

Ustekinumab is a humanized mAb that binds to the p40 subunit of IL-12/IL-23 cytokines. These molecules have a pivotal role in the development of Th1 and Th17 cells [192]. Trials have demonstrated that ustekinumab reduces the infiltration of T lymphocytes, dendritic cells, and mast cells in the skin [172]. However, no meaningful clinical efficacy has been reported [193,194]. 

### 14.3. Tralokinumab

Tralokinumab is a recombinant IgG4 neutralizing mAb that binds to IL-13 and interferes with the interaction between IL-13 and its receptor [195]. In trials, the drug has not shown any particular difference from placebo [196]. However, various studies are still ongoing [172]. 

### 14.4. Lebrikizumab

Lebrikizumab is another IL-13 binding mAb, but its efficacy and safety profile have been thought to be different from other drugs that bind IL-13. Initial results on the drug’s efficacy have shown superior results compared to placebo with repeated doses [197] and no significant adverse effect has been reported [197]**. **

### 14.5. Antithymic stromal lymphopoietin receptor (TSLPR)

TSLP is an IL-7–like cytokine that is primarily expressed in epithelial cells, keratinocytes, and ocular cells, and has a role in allergic inflammatory disorders [198–200]. Secretion of the cytokine can be stimulated by allergens, viruses, trauma, and smoke [201]. Myeloid dendritic cells (mDCs) are activated by TSLP, and begin a cascade of events that lead to the differentiation of naive T cells into Th2 lymphocytes [202]. Blocking of TSLPR was first explored in asthma treatment [203]; other trials comprised of patients with AD have also begun [196]. TSLP’s role in the exacerbation of pruritus via sensory neurons has been demonstrated [90]. Studies on AD have revealed significant improvement in the severity scores of the patients [196].

### 14.6. Phosphodiesterase (PDE) inhibitors

In the 1980s, enhanced PDE activity was determined in blood cells as well as cord blood cells of patients with AD [204]. Since then, the effects of PDE inhibitors in patients with AD have been demonstrated [205]. Topical forms of PDE inhibitors are crisaborole, OPA-15406, RVT-501, and roflumilast, while systemic (oral) forms of the drug are apremilast and roflumilast [204]. In December 2016, FDA approved crisaborole for the treatment of mild-to-moderate atopic dermatitis in patients aged ≥2 years [204]. 

## 15. Burden of atopic dermatitis

AD mostly begins in childhood and may persist throughout adulthood [206]. Therefore, it may have both economic and psychosocial impacts on patients and their families [207]. Evaluation of quality of life (QoL), social, academic, and occupational impacts, and financial costs are important in capturing the whole picture when assessing the burden of AD. Patients with AD were shown to have lower overall QoL [208], especially in the form of mental health and social function [208]. A study by Solomon et al. showed that children with AD had severe sleep difficulties and were more dependent on their caregivers than patients with other chronic disorders [209]. Chamlin et al. also showed that these children were avoided by other people, causing social isolation [210]. Many studies have shown that sleep deprivation, isolation from social activities, and feelings of embarrassment due to itching and appearance of the eczematous lesions were the major problems faced by children with AD [211,212]. In a study performed on children and adults with AD, it was reported that 87.1% of patients had difficulties falling asleep, and 73.5% reported that severe itching was the cause for waking up [213].

AD also affects parents. The parents of children with AD also reported sleep disturbance [210] and stress [214], and were found to spend a significant amount of time on daily skin care [210] and hygiene [215]. Furthermore, the time spent by parents with the siblings of children with AD was also reported to be decreased [216], suggesting that AD has detrimental effects on the entire family. Lifestyle changes including modified dietary habits and usage of cleaning products and moisturizers for the skin in patients with AD were also shown to cause a substantial burden on the family budget [217]. Additionally, regular physician visits may cause loss of worktime for parents, while spending excessive time and effort on the care of a child with AD may also cause social isolation for the parents [217]. 

Assessing the exact financial burden of AD on the family budget and the health system is very difficult due to the indirect costs as well as direct expenditures. Additionally, the total costs of AD are also dependent on disease severity and may differ significantly from country to country [218]. Indirect costs include loss of labor and decrease in productivity at work [207]. Direct costs consist of pharmacy expenditures, follow-up visits, applications to emergency departments, and spending on special diet programs [207]. A striking result reported by Storan et al. indicated that 86% of the children admitted to their inpatient clinic were diagnosed with AD [219]. This study demonstrates the financial strain caused by AD on the health system. According to studies on economic costs of chronic diseases, AD was in fourth place among skin diseases [220]. In 2004, the annual cost of AD was 4.228 billion dollars in the United States [220], and had increased to approximately 5.297 billion dollars in 2015 [207]. Although indirect costs are not exactly estimated, the direct costs of AD have increased significantly over the years. 

## 16. Future perspectives 

The complexity of AD pathogenesis, the lack of a definitive treatment, and the fact that available therapies only provide transient well-being are among the factors that fuel the research for a cure for AD. Mainly due to this enthusiasm, experts have explored many novel molecules, mutations, and genes responsible for the disease. New treatment modalities are reported to be promising for the treatment of AD. However, it is also important to consider that current treatments (and the majority of proposed treatments) do not aim to prevent the development of atopic march. For this reason, researchers on this field concentrate not only on antiinflammatory drugs, but also on immunological and biological agents. With our knowledge today, it is evident that AD has a significant immunological background, and if the immunological dysregulation underlying the disease can be elucidated and targeted in treatment, success in AD treatment and prevention will be achieved. Pruritus is one of the most important problems of AD and suggested treatments must address this problem. Pruritus causes exhaustion, despair, and sleep deprivation in patients. Unfortunately, current drugs are insufficient for the control of pruritus. Therefore, development of antipruritic drugs with higher effectiveness should be regarded as high priority. The current therapeutic approach to AD, the advantages of treatments, and unmet needs are summarized in Table 2.

**Table 2:** Current treatment of AD, advantages, and unmet needs.

Current treatment	Unmet needs	New goals
Topical and systemic corticosteroids, calcineurine inhibitors, immunomodulators	Disease flare-ups are seen despite long term usage of today’s available drugs	Minimizing flare-ups, achieving disease free life
Drugs are used for months or years in general	Long term application may not be safe in children (malignancy in calcineurine inhibitors)	Patients should adapt therapy easily in their lives, attaining short term using drugs
Only for eczematous lesions, providing temporary wellbeing, not prevent recurrence and other allergic diseases	Generally proceeds to asthma and allergic rhinitis	Prevention of other allergic disorders with radical treatment strategies
Systemic antihistamines are used for pruritus and give sedation	Pruritus does not cease and is recalcitrant, psychological exhaustion appears frequently due to pruritus	Effective antipruritic drugs
Parents and patients should spend many hours in a day for skin care. In addition, moisturizers and drugs are expensive	Time-consuming and economic burden on families and country economy	Spending shorter time for skin care, cheap and effective alternative treatment options

## 17. Conclusion 

The aims of new therapies for AD are not only to procure a definite treatment but also to enhance the adherence of patients and parents to the therapy [221]. Other factors to consider as treatment goals in AD are the prevention of secondary infections, providing support to patients with psychological problems, and increasing the awareness of the population and physicians to a disease with severe consequences on the patient, family, and country.
